# Can a Positive Allosteric Modulation of GABAergic Receptors Improve Motor Symptoms in Patients with Parkinson's Disease? The Potential Role of Zolpidem in the Treatment of Parkinson's Disease

**DOI:** 10.1155/2016/2531812

**Published:** 2016-05-17

**Authors:** Antonio Daniele, Francesco Panza, Antonio Greco, Giancarlo Logroscino, Davide Seripa

**Affiliations:** ^1^Institute of Neurology, Catholic University of the Sacred Heart, 00168 Rome, Italy; ^2^Geriatric Unit and Laboratory of Gerontology and Geriatrics, Department of Medical Sciences, IRCCS “Casa Sollievo della Sofferenza”, San Giovanni Rotondo, 71013 Foggia, Italy; ^3^Neurodegenerative Disease Unit, Department of Basic Medicine, Neuroscience, and Sense Organs, University of Bari Aldo Moro, 70121 Bari, Italy; ^4^Department of Clinical Research in Neurology, University of Bari Aldo Moro, “Pia Fondazione Cardinale G. Panico”, Tricase, 73039 Lecce, Italy

## Abstract

At present, patients with advanced Parkinson's disease (PD) are unsatisfactorily controlled by currently used anti-Parkinsonian dopaminergic drugs. Various studies suggest that therapeutic strategies based on nondopaminergic drugs might be helpful in PD. Zolpidem, an imidazopyridine widely used as sleep inducer, shows high affinity only for GABA_A_ receptors containing the *α*-1 subunit and facilitates GABAergic neurotransmission through a positive allosteric modulation of GABA_A_ receptors. Various observations, although preliminary, consistently suggest that in PD patients zolpidem may induce beneficial (and sometimes remarkable) effects on motor symptoms even after single doses and may also improve dyskinesias. Since a high density of zolpidem binding sites is in the two main output structures of the basal ganglia which are abnormally overactive in PD (internal globus pallidus, GPi, and substantia nigra pars reticulata, SNr), it was hypothesized that in PD patients zolpidem may induce through GABA_A_ receptors an inhibition of GPi and SNr (and, possibly, of the subthalamic nucleus also), resulting in an increased activity of motor cortical areas (such as supplementary motor area), which may give rise to improvement of motor symptoms of PD. Randomized clinical trials are needed in order to assess the efficacy, safety, and tolerability of zolpidem in treating motor symptoms of PD.

## 1. Introduction

Parkinson's disease (PD) is the most common neurodegenerative movement disorder, with a prevalence of 0.3% in the general population in industrialized countries and a prevalence of 4% in elderly people aged over 80 years [1]. The clinical manifestations of PD include motor symptoms (bradykinesia, rigidity, postural instability, and resting tremor) and a variety of nonmotor symptoms, including cognitive impairment, behavioural symptoms [2], sleep disturbances, and olfactory and autonomic dysfunction.

At present, pharmacological treatment of motor symptoms of PD, mainly based on the administration of dopaminergic drugs (levodopa, dopamine agonists) and other drugs involved in levodopa metabolism (monoamine oxidase-B inhibitors, COMT inhibitors), may induce beneficial effects on Parkinsonian motor symptoms only for some years after the onset of such symptoms and becomes usually less effective later in the disease course [3]. In late disease stages, various complications usually arise in PD patients treated by dopaminergic drugs, such as* on-off* fluctuations of Parkinsonian symptoms and dyskinesias (involuntary movements of the head, trunk, and upper or lower limbs). On the whole, at present, most patients with advanced PD are unsatisfactorily controlled by the currently most widely used anti-Parkinsonian drugs.

In the last fifteen years, the most significant advances in the treatment of PD were represented by neurosurgical procedures aimed at modulating the activity of specific brain structures, such as deep brain stimulation (DBS) of the internal globus pallidus (GPi) and DBS of the subthalamic nucleus (STN), which may induce remarkable beneficial effects in PD patients [4], even after more than 10 years from the neurosurgical intervention [5]. Preliminary studies suggest that neurosurgical procedures alternative to DBS, such as extradural stimulation of the motor cortex, are definitely less effective than DBS of the STN or GPi, although extradural stimulation of the motor cortex may induce slight beneficial effects on Parkinsonian motor symptoms, including axial motor symptoms which are usually poorly responsive to drug treatment [6, 7].

It is well established that neuropathological processes underlying PD involve not only dopaminergic systems, but also noradrenergic, serotonergic, glutamatergic, and cholinergic systems in several brain structures. So far, a number of nondopaminergic drugs have been available for the treatment of motor symptoms of PD [8], while other nondopaminergic drugs are still under investigation. Among anti-Parkinsonian drugs with pharmacological effects on nondopaminergic systems, adenosine A2A receptor antagonists, safinamide (an agent inhibiting both glutamate release and monoamine oxidase-B), and the antiepileptic agent zonisamide have been proposed as add-on drugs which may extend the duration of effects of levodopa. To reduce levodopa-induced dyskinesias, antiglutamatergic drugs, such as amantadine (a nonselective N-methyl D-aspartate antagonist) and antagonists of metabotropic glutamate receptor (mGluR_5_), might be helpful in PD patients. As regards drugs with pharmacological effects on serotonergic and noradrenergic systems, 5-HT2A/2C antagonists (such as the atypical antipsychotic clozapine) and *α*2-adrenergic receptor antagonist (such as fipamezole) may reduce dyskinesias in PD patients. As to nondopaminergic drugs which can be used in PD patients for the treatment of tremor, various options can be considered, including anticholinergic drugs (muscarinic M4 cholinergic antagonists), *β*-adrenergic antagonists (such as propranolol), 5-HT2A antagonists, and drugs with pharmacological effects on both serotonergic and cholinergic systems (such as clozapine and the antidepressant mirtazapine). It has been pointed out that while dopaminergic drugs still remain the most effective option to treat motor symptoms in PD, alternative therapeutic strategies based on the use of nondopaminergic drugs in PD are currently needed to improve symptoms that are (or become) poorly responsive to dopaminergic drugs [8].

## 2. Potential Beneficial Effects of Zolpidem, a Positive Allosteric Modulator of GABA_**A**_ Receptors, on the Motor Symptoms of PD

In the late 1980s, some opinion leaders in the field of movement disorders [9] had suggested that drugs that may specifically enhance GABAergic neurotransmission may be potentially helpful in the treatment of PD, but at that time there was scanty evidence supporting this intriguing hypothesis. In particular, a pilot study had suggested that GABA agonists such as progabide [10] might be helpful in the treatment of motor symptoms of patients with PD, in agreement with an experimental study in rats showing the effects of progabide [11] on motor function (turning behaviour) and with previous postmortem findings showing abnormalities in the GABAergic systems of the brain of patients with Parkinson's disease [12].

Zolpidem is an imidazopyridine drug with short half-life, which has been widely used for more than two decades for the treatment of insomnia. Both zolpidem and benzodiazepines (BZ) exert their pharmacological effects by facilitating GABAergic neurotransmission through a positive allosteric modulation of GABA_A_ receptors. GABA_A_ receptors, which are the major inhibitory neurotransmitter receptors in the brain, are heteropentamers and are classified according to their *α* subunits. In GABA_A_ receptors containing specific isoforms of *α* subunits (*α*1, *α*2, *α*3, and *α*5), there is a binding site for BZ and zolpidem in the *α* subunit [13]. BZ bind relatively unselectively to several subtypes of GABA_A_ receptors, namely, those containing the *α*1, *α*2, *α*3, or *α*5 subunits. Unlike BZ, zolpidem is an agonist with high affinity only for GABA_A_ receptors containing the *α*-1 subunit [14]. The interaction of zolpidem with the BZ binding site of GABA_A_ receptors (particularly, of GABA_A_ receptors containing the *α*-1 subunit, given the high affinity of zolpidem for such GABA_A_ receptor subtype) results in facilitation of GABAergic neurotransmission, particularly in neurons with GABA_A_ receptors containing the *α*-1 subunit. It has been recently suggested that, according to pharmacological data from both rodents and nonhuman primates, among drugs currently available for clinicians, zolpidem has a peculiar pharmacological profile, which differentiates zolpidem from BZ [15]. Some studies have previously suggested that there is a high density of zolpidem binding sites in the GPi and in the substantia nigra pars reticulata (SNr), which are the output structures of the basal ganglia [16, 17].

In the mid-1990s, Daniele and coworkers observed a 61-year-old woman affected by juvenile-onset PD in an advanced disease stage after a 25-year clinical history, who received at bedtime an oral immediate-release formulation of zolpidem for insomnia [18]. Surprisingly, 45 to 60 minutes after the assumption of the first 10 mg dose of zolpidem as sleep inducer, this patient showed no drowsiness but a remarkable improvement of Parkinsonian motor symptoms (akinesia, rigidity, and resting tremor). In this woman, the motor improvement induced by a single 10 mg dose of zolpidem immediate-release was comparable to the improvement observed after the administration of levodopa/carbidopa, while no beneficial effect on her Parkinsonian motor symptoms was observed when she assumed other hypnotic drugs (triazolam and zopiclone). This patient with advanced PD assumed zolpidem chronically (10 mg four times daily) for more than 5 years, with no side effects and persistent beneficial effects on Parkinsonian motor symptoms over time.

Following such serendipitous observation, a pilot study (double-blind, placebo-controlled crossover) was carried out in ten patients with a clinical diagnosis of PD [18], with a mean disease duration of 9.2 years and a mean Hoen and Yahr score of 2.9 years. Since in the first serendipitous observation even a single 10 mg dose of zolpidem showed beneficial effects on Parkinsonian motor symptoms about one hour after the drug administration, in such pilot study PD patients were given in randomized order a 10 mg fixed dose of immediate-release zolpidem and placebo in two different days, after withholding of all anti-Parkinsonian medication 12 hours before motor assessment. PD patients were assessed by means of the motor examination part (part III) of the Unified Parkinson's Disease Rating Scale (UPDRS-III) before (baseline) and one hour after the administration of zolpidem or placebo. In each individual PD patient, a positive response was defined as a decrease of more than 20% of the UPDRS-III score at baseline. In this pilot study, the administration of a single 10 mg fixed dose of immediate-release zolpidem, unlike placebo, induced in the overall group of ten PD patients a statistically significant improvement of Parkinsonian motor symptoms, with a 18% mean reduction of UPDRS-III scores from baseline. Six out of 10 PD patients could be defined as responders to a single 10 mg dose of zolpidem and showed on average a 30% improvement of motor symptoms, ranging from 21% to 59% in different individuals affected by PD. In PD patients who resulted in being responders to zolpidem, such improvement (which appeared 45–60 minutes after the administration of zolpidem and lasted about 2–4 hours) mainly involved facial expression, rigidity, akinesia/bradykinesia, posture, and gait. In this group of 10 PD patients, in those patients who presented resting tremor, the administration of a single 10 mg dose of zolpidem improved also resting tremor, although this finding was not mentioned among the results reported by Daniele and coworkers [18]. Interestingly, the 6 patients who resulted in being responders to zolpidem included 3 out of the 4 most severely affected PD patients and 3 out of the 6 less severely affected PD patients, suggesting the hypothesis that beneficial effects of a single 10 mg dose of immediate-release zolpidem on Parkinsonian motor symptoms may perhaps be more likely observed in PD patients with more severe motor symptoms. In this pilot study, the only adverse effect observed after the administration of a 10 mg single dose of immediate-release zolpidem was drowsiness, which occurred in 4 out of 10 PD patients and was severe in 2 patients, moderate in one patient, and mild in another patient. Interestingly, drowsiness was observed only in one out of the 4 most severely affected patients and 3 out of the 6 less severely affected patients, suggesting that after a single 10 mg dose of immediate-release zolpidem the occurrence of drowsiness may be less likely observed in PD patients with more severe motor symptoms. Despite the beneficial effects on Parkinsonian motor symptoms in 6 out of 10 PD patients, a single 10 mg dose of zolpidem did not induce dyskinesias in any patient, even in 3 PD patients who had showed levodopa-induced dyskinesias.

In 2000, Růžička and coworkers [19] reported a 45-year-old woman with 12-year history of PD. Eight months after levodopa was started in 1992 and 5 years after disease onset, the patient developed choreic dyskinesias of the right hand. Later on, dyskinesias became generalized and were present most of the time. A number of treatment strategies (controlled-release levodopa, addition to levodopa of bromocriptine, terguride, and tiapride) failed to alleviate such dyskinesias. In 1994, she was given zolpidem for insomnia, and 30 minutes after 2.5 to 5 mg of zolpidem, her dyskinesias substantially diminished or disappeared for 2 hours, without any drowsiness. In 1997, when motor fluctuations developed, she also noticed an improvement of motor function, if the dose of zolpidem was taken in the “off” state. This PD patient increased the dosage of zolpidem up to 30 mg per day (divided into 6 doses of 5 mg), with a persistent antidyskinetic effect “on” levodopa and improved motricity (especially gait initiation) in the “off” state, without daytime somnolence. After more than 3 years of chronic treatment with zolpidem, the single doses of zolpidem had not been substantially increased. This observation suggested the potential benefits of the chronic administration of zolpidem not only on Parkinsonian motor symptoms, but also on dyskinesias in late stages of PD.

In 2004, at the 8th International Congress of Parkinson's Disease and Movement Disorders organized by the Movement Disorders Society, Tagaris and coworkers [20] presented preliminary data on some remarkable beneficial effects induced by the administration of zolpidem on motor symptoms of patients with advanced PD. As far as we are aware, such interesting data have been reported so far only as an abstract of such congress. In an initial study on a group of 14 PD patients receiving zolpidem for insomnia ([20]; Tagaris, personal communication, 2004), Tagaris and coworkers investigated a subgroup of 5 PD patients, who self-reported robust beneficial effects on Parkinsonian motor symptoms induced by the assumption of zolpidem for insomnia. In this first subgroup of 5 PD patients, who also showed frequent and severe dyskinesias, Parkinsonian motor symptoms were assessed by means of the UPDRS-III before and after the administration of single doses of levodopa and single 5 or 10 mg doses of immediate-release zolpidem. In this subgroup of 5 PD patients, zolpidem (unlike the BZ temazepam) induced a dramatic improvement of Parkinsonian motor symptoms, comparable to the improvement induced by levodopa. Accordingly, in this subgroup of 5 PD patients, zolpidem was administered chronically, in daily doses of 30 mg or more. One of such PD patients out of five showed a tendency to abuse zolpidem and assumed zolpidem almost every 2 hours. Interestingly, 2 out of 5 PD patients reported an improvement of levodopa-induced dyskinesias if levodopa was administered in association with zolpidem, while no patient reported dyskinesias after the administration of zolpidem. In a second study (Tagaris, personal communication, 2004 [20]), a single oral dose of immediate-release zolpidem 5 mg or flunitrazepam 1 mg was administered to another group of 10 patients with advanced PD, who had never received zolpidem before. If no anti-Parkinsonian effect and no sleepiness were detected after the administration of a 5 mg single dose of zolpidem (as observed in 5 out of 10 PD patients), the single dose of zolpidem was then increased to 10 mg. Soon (30–45 minutes) after the administration of a single oral dose of zolpidem, a significant motor improvement (decrease of more than 20% in the baseline score of UPDRS) was observed in 7 out of 10 patients with advanced PD. In these patients, no improvement of Parkinsonian motor symptoms was observed after the administration of the BZ flunitrazepam. In this latter study, a slight somnolence was observed in 4 out of those 5 PD patients who were given a single 10 mg dose of zolpidem and in 2 out of those 5 PD patients who were given a single 5 mg dose of zolpidem, while sleepiness was observed only in 1 PD patient after a single 5 mg dose of zolpidem. These preliminary observations showed that single oral doses of zolpidem as well as chronic administration of zolpidem may improve Parkinsonian motor symptoms in patients with advanced PD, suggesting that the anti-Parkinsonian effects of zolpidem may perhaps be more pronounced in PD patients with dyskinesias.

The hypothesis that the administration of zolpidem is more likely to improve Parkinsonian motor symptoms in patients with advanced PD was supported by a clinical observation reported by Chen and coworkers [21] in a 53-year-old patient with advanced PD, who underwent unilateral pallidotomy, unilateral thalamotomy, and bilateral DBS of the STN. In this PD patient, the administration of a single 10 mg dose of zolpidem rapidly induced a remarkable amelioration of Parkinsonian motor symptoms (akinesia, dystonia, and dyskinesias), with a 27% improvement on the UPDRS-III scale and no significant somnolence with a 10 mg dose of zolpidem. After the administration of a lower dosage of zolpidem (5 mg), there was an amelioration of dystonia and dyskinesias, in the absence of a satisfactory improvement of other Parkinsonian motor symptoms. In this patient, zolpidem was chronically administered at a daily dosage of 25 mg (5 mg three times a day and 10 mg before sleeping). This observation suggests that, in patients with advanced PD, such as patients who underwent DBS of the STN and more invasive neurosurgical interventions (pallidotomy and thalamotomy), zolpidem may improve various Parkinsonian motor symptoms, including dystonia and dyskinesias, possibly at relatively lower doses.

In 2010, at the 14th International Congress of Parkinson's Disease and Movement Disorders organized by the Movement Disorders Society, Abaroa and coworkers [22] described three patients affected by PD with motor fluctuations and dyskinesias. The first patient was a 50-year-old man, with a 3-year disease duration, in stage 3 on the Hoehn and Yahr (HY) scale. He showed wearing off and dyskinesias, with foot dystonia and severe pain in lower limbs in off state. The second patient (a 76-year-old man, with a 12-year disease duration, in stage 3 on the HY scale) showed wearing off and severe off states with akinesia, foot and oromandibular dystonia, and pain in lower limbs. The third patient (a 61-year-old woman, with an 8-year disease duration, in stage 3 on the HY scale) showed wearing off, dyskinesias, severe trunk, and cervical dystonia associated with pain in lower limbs in off state. All 3 patients were assessed by means of the UPDRS-III before and after the oral administration of a single 7.5 mg dose of zolpidem. About 15–20 minutes after the oral administration of such single dose of zolpidem, in all patients dystonia disappeared and, for about 1-2 hours, there was a remarkable improvement of pain, dysphagia, and other Parkinsonian motors symptoms assessed by UPDRS-III (with a reduction of 30% or more of UPDRS-III scores). None of the 3 PD patients showed drowsiness after the assumption of zolpidem. It was briefly mentioned [22] that in two PD patients the remarkable improvement of UPDRS-III scores obtained after the administration of a single dose of zolpidem was comparable to the improvement obtained with levodopa, while in a third patient the improvement of UPDRS-III scores obtained after the administration of a 7.5 mg dose of zolpidem was even greater than the motor improvement obtained with levodopa. This report suggests that zolpidem may improve Parkinsonian motor symptoms (including dystonia and dyskinesias) in patients with fluctuating PD, such as patients with mild to moderate bilateral disease who have complications associated with long-term levodopa treatment.

In 2012, Huang and coworkers [23] reported a 61-year-old housewife with PD and a 12-year disease duration, with rest tremor of right limbs as presenting symptom. The patient was referred to hospital in March 2009 because of both the shortening of beneficial effects of pharmacological treatment in the preceding 3 years and the appearance of on-phase dyskinesias in the preceding 2 years. In June 2009, she underwent bilateral DBS of STN. The day after the neurosurgical intervention, the patient started to show “fluctuating spells of mental confusion (inertia and confusion with incoherent speech and fearing of being killed),” which persisted over time. In July 2009, soon after the administration of zolpidem for insomnia, the patient showed for few hours an improvement of Parkinsonian motor symptoms (with approximately a 25% improvement on the UPDRS-III scale) and an amelioration of behavioural symptoms: “she could chat normally with her caregiver and walk with assistance after taking zolpidem.” In keeping with the behavioural improvement observed after the administration of zolpidem, the total score on the Neuropsychiatric Inventory (which was administered to assess her behavioural symptoms) decreased from 56 to 30. Accordingly, zolpidem was administered to the patient at a dosage of 10 mg three times per day. In this patient, positron emission tomography with ^18F^F-fluorodeoxyglucose showed a reduction of metabolism in the right frontal and parietal cortex and in caudate nucleus, which improved after the administration of zolpidem.

In recent years, neurophysiological studies aimed at registering cerebral electrical activity in PD showed that in patients with PD a pathological increase of beta activity (15–35 Hz) oscillations may occur in various brain structures, such as the STN and the cerebral cortex [24, 25]. A relationship between such pathologically increased beta activity in PD patients and severity of Parkinsonian motor symptoms has been hypothesized, since a reduction of such pathologically increased beta activity may be observed in PD patients after improvement of Parkinsonian motor symptoms following either pharmacological treatment with levodopa [26] or DBS of the STN [27]. It has been also suggested that beta activity oscillations in the primary motor cortex may be modulated by GABAergic mechanisms and that drugs modulating GABA_A_ receptors such as diazepam may reduce the frequency of beta activity oscillations [28].

In a recent study, Hall and coworkers [29] investigated a group of 9 PD patients in early disease stages (with unilateral Parkinsonian motor symptoms only) and a group of 9 age-matched healthy controls. Participants of both groups underwent two magnetoencephalography (MEG) recording sessions, at baseline and after the administration of a single low (namely, subsedative) oral dose of zolpidem (0.05 mg/kg). At baseline, as shown in previous studies, PD patients showed an increase of beta power in the primary motor cortex (M1), which was greater in M1 area contralateral to the body side affected by Parkinsonian motor symptoms, as compared to M1 area ipsilateral to the body side affected by Parkinsonian motor symptoms. After the administration of a single low (individually adjusted, according to body weight) oral dose of zolpidem, all PD patients showed a remarkable and statistically significant improvement of Parkinsonian motor symptoms (with approximately a 50% mean reduction of total score on UPDRS-III), with a statistically significant amelioration of most items of UPDRS-III (resting and postural tremor, rigidity of upper and lower limb, finger tapping, hand movements, rapid alternating movements of hands, postural stability, and bradykinesia). Moreover, after the administration of a single low oral dose of zolpidem in PD patients, beta power was significantly reduced in contralateral M1 area, while it was significantly increased in ipsilateral M1 area, resulting in a hemispheric beta power ratio between the right and left M1 areas that approached parity. Furthermore, in PD patients there was highly significant correlation between the changes in hemispheric beta power ratio in M1 areas induced by zolpidem (significant reduction of beta power in contralateral M1 area and significant increase of beta power in ipsilateral M1 area, resulting in a hemispheric beta power ratio between the right and left M1 areas that approached parity) and the improvement induced by zolpidem on Parkinsonian motor symptoms as assessed by scores on UPDRS-III scale. These findings show that in PD patients the oral administration of zolpidem in low doses may result in an improvement of Parkinsonian motor symptoms, which is associated with both a reduction of beta power in contralateral M1 area and an increase of beta power in ipsilateral M1 area, thus rebalancing the dynamic range of M1 network oscillations between M1 areas of the two cerebral hemispheres [29]. From a clinical viewpoint, this study suggests that low doses of zolpidem may improve Parkinsonian motor symptoms also in PD patients in early disease stages. It has been hypothesized that zolpidem may markedly reduce the synchronous power of pathological oscillations in brain structures in which a pathologically increased beta power can be detected, namely, that zolpidem may desynchronize such pathological activity, with subsequent improvement of motor function [30].

In conclusion, the limited evidence available so far suggests that, after the administration of single oral doses mostly ranging between 2.5 mg and 10 mg, a response (motor improvement) to immediate-release formulations of zolpidem might be more likely in PD patients with the following clinical features: (a) patients with moderate to severe PD [18], including patients with advanced PD [18, 20, 21, 23]; (b) PD patients with dyskinesias [19–22] and motor fluctuations [19, 22]. Such preliminary evidence, however, does not rule out the possibility that even patients with mild PD may respond to zolpidem, as it has been shown that PD patients in early disease stages [22, 29] may show an improvement of Parkinsonian motor symptoms after the administration of low (0.05 mg/kg) single oral doses of zolpidem (e.g., a 3.5 mg dose for a patient who weighs 70 kg).

## 3. Possible Mechanisms Underlying the Potential Beneficial Effects of Zolpidem on Parkinsonian Motor Symptoms

**Figure 1:**
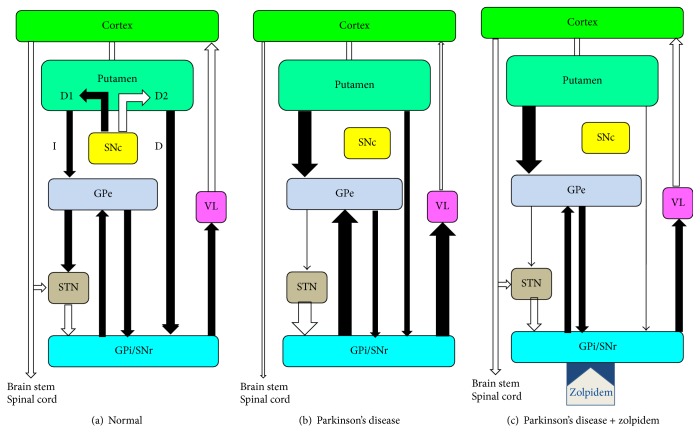
Schematic representations of the activity of the cortical-basal ganglia loops, showing the direct (D) and indirect (I) circuits in normal subjects (a), patients with Parkinson's disease/Parkinsonism (b), and patients with Parkinson's disease treated with zolpidem (c). In PD [18], zolpidem may induce, through GABA_A_ receptors containing the *α*1 subunit, a selective inhibition of the GPi and the SNr (the two main inhibitory GABAergic output structures of the basal ganglia), which are overactive in PD (b) and show binding sites for zolpidem (c). The inhibition of the GPi and the SNr directly induced by zolpidem may result in an increased activity of some motor cortical areas (such as supplementary motor area), which may underlie the possible improvement of Parkinsonian motor symptoms which can be observed after the administration of zolpidem. Although binding sites for zolpidem in the STN are not displayed in (c), the STN was found to have binding sites for zolpidem [36] and might be reasonably considered an additional target for zolpidem in PD patients. In PD, zolpidem might inhibit the STN as well, giving rise to decreased excitatory inputs from the STN to the GPi and SNr, resulting in a further mechanism leading to a decreased activity of the GPi and SNr, indirectly induced by zolpidem through its inhibition of the STN. White arrows = excitatory connections; black arrows = inhibitory connections.

It is well established that dopaminergic depletion resulting from neurodegenerative processes affecting dopaminergic neurons in the SN pars compacta gives rise in PD patients to overactivity of the two main inhibitory output structures of the basal ganglia, namely, the GPi and the SNr. The overactivity of such two inhibitory output GABAergic structures in the basal ganglia gives rise to decreased activity in specific thalamic nuclei and, in turn, in reduced activity of motor cortical areas (such as supplementary motor area), which may result in motor symptoms of PD (Figure 1(b)).

In order to explain the quite surprising potential beneficial effects on Parkinsonian motor symptoms even of single doses of the hypnotic drug zolpidem observed in their pilot study, Daniele and coworkers [18] proposed a hypothesis about the possible mechanisms underlying these effects. Such hypothesis is based on the fact that zolpidem, unlike benzodiazepines, is an agonist with high affinity for GABA_A_ receptors containing the *α*-1 subunit [14], previously named GABA_A_-BZ_1_ receptors, and therefore facilitates GABAergic neurotransmission in brain structures which preferentially contain GABA_A_ receptors with the *α*-1 subunit. Since some studies [16, 17] had suggested that there is a high density of zolpidem binding sites in the GPi and in the SNr (the two main output structures of the basal ganglia, which are abnormally overactive in PD), it was hypothesized that in PD patients zolpidem may induce through GABA_A_ receptors a selective inhibition of such two overactive and inhibitory GABAergic structures (the GPi and the SNr), resulting in turn in an increased activity of motor cortical areas (such as supplementary motor area), which may underlie the possible improvement of Parkinsonian motor symptoms which can be observed after the administration of zolpidem (Figure 1(c)) in PD [18] and other Parkinsonian syndromes [31]. According to this hypothesis, it was also speculated that the administration of zolpidem could provide a pharmacological equivalent of posteroventral pallidotomy [18].

This hypothesis has been subsequently supported by experimental studies in rats [32, 33] and has been quoted in several publications as a plausible explanation for the possible anti-Parkinsonian effects of zolpidem in patients with PD and other Parkinsonian syndromes [19–22, 31, 34, 35].

Chen and coworkers [32] investigated the* in vitro* and* in vivo* effects of zolpidem on the globus pallidus (GP) in rats. In their experimental study on* in vitro* slices of the GP in rats, a single 100 nM dose of zolpidem enhanced the action of GABA on postsynaptic GABA_A_ receptors and prolonged the duration of inhibitory postsynaptic currents recorded from neurons of the GP. Moreover, in a study aimed at investigating the effects of zolpidem* in vivo* on the GP in rats [32], when a single dose of zolpidem was acutely microinjected into the GP unilaterally, a change in motor behaviour, namely, a robust ipsilateral rotation (turning behaviour), was observed in the behaving rats. Chen and coworkers [32] suggested that this motor effect of zolpidem* in vivo* was due to the inhibition of the activity of neurons of the GP induced by zolpidem. All such effects of zolpidem,* in vitro* and* in vivo*, were sensitive to the benzodiazepine antagonist flumazenil. These findings on the* in vitro* and* in vivo* effects of zolpidem on the GP in rats provided experimental support to the hypothesis of a GP-mediated mechanism of the anti-Parkinson effects of zolpidem in PD and suggested the need of further investigations aimed at assessing the potential beneficial effects of zolpidem in the treatment of movement disorders originating from dysfunction of the basal ganglia.

Zhang and coworkers [33] investigated* in vitro* and* in vivo* the effects of zolpidem on the SNr in rats. In* in vitro* slices of the SNr, superfusion of zolpidem at 100 nM/L induced a GABAergic inhibition of the SNr by activating postsynaptic GABA_A_ receptors and prolonged the duration of inhibitory postsynaptic currents recorded from neurons of SNr. In an experimental study on the effects of zolpidem* in vivo* on the SNr, it was observed that a unilateral microinjection of zolpidem into the SNr caused a change in motor behaviour, namely, a robust contralateral rotation in the behaving rats. All such effects of zolpidem,* in vitro* and* in vivo*, were sensitive to the BZ antagonist flumazenil. These findings on the* in vitro* and* in vivo* effects of zolpidem on the SNr provided experimental evidence about a possible SNr-mediated mechanism of the anti-Parkinson effects of zolpidem in PD and further supported the idea of a potential role of zolpidem in the treatment of movement disorders arising from dysfunction of the basal ganglia.

In conclusion, the two studies mentioned above [32, 33] provided an experimental support to the hypothesis of Daniele and coworkers [18], who suggested that the potential beneficial effects of zolpidem on the motor symptoms of PD patients may fundamentally arise from a selective pharmacological inhibition, induced by zolpidem through GABA_A_ receptors in GPi and SNr (Figure 1(c)), of such overactive inhibitory structures (GPi and SNr), resulting in an increased activity of specific thalamic nuclei and, in turn, in an increased activity of specific motor cortical areas (such as supplementary motor area), which may give rise to an improvement of Parkinsonian motor symptoms.

Further data about the possible mechanisms which may underlie the potential anti-Parkinsonian effects of zolpidem were provided by an additional experimental study in rats [36], suggesting that zolpidem may induce beneficial effects on the motor symptoms of patients with PD also by inhibiting at least another subcortical nucleus overactive in PD (besides GPi and SNr), namely, the STN. Chen and coworkers [36] carried out an* in vitro* and* in vivo* study on the effects of zolpidem on the STN, a brain structure which also shows a high density of zolpidem binding sites [17] and plays a key role in the indirect pathway of the basal ganglia circuits. In the* in vitro* study [36], whole-cell patch clamp recordings were used to investigate the modulation of zolpidem on GABA_A_ receptor-mediated inhibitory synaptic currents in the STN. Zolpidem at a 100 nM dose significantly prolonged the decay time and rise time of miniature inhibitory postsynaptic currents in the STN, with no effect on the amplitude and frequency. At a high concentration of 1 *µ*M, zolpidem significantly increased the decay time, rise time, amplitude, and frequency of miniature inhibitory postsynaptic currents in the STN. The BZ antagonist flumazenil could completely block the effects induced by zolpidem, confirming that the effects of zolpidem are mediated by the BZ recognition site. In an* in vivo* study [36], a unilateral microinjection of* zolpidem* into the STN induced a significant contralateral rotation in the behaving rats. These results about the* in vitro* and* in vivo* effects of zolpidem on the STN provided experimental support to the hypothesis of a possible STN-mediated mechanism of the anti-Parkinson effects of zolpidem in PD and were in keeping with previous suggestions about a potential role of zolpidem in the treatment of movement disorders associated with a dysfunction of the basal ganglia. According to these latter findings, we might hypothesize that the administration of zolpidem, to some extent and perhaps in a subset of PD patients, could maybe provide a pharmacological equivalent also of neurosurgical procedures aimed at inhibiting the overactivity of the STN in patients with PD, such as DBS of the STN.

In conclusion, current available evidence suggests that zolpidem may induce a selective inhibition not only of the two output structures of the basal ganglia (GPi and SNr) which are overactive in PD [18], but also of an additional overactive structure in the basal ganglia pathways such as the STN, which might be reasonably considered an additional target for zolpidem, although it is not reported in Figure 1(c) as a target structure for zolpidem. Accordingly, zolpidem may induce a selective inhibition of various overactive structures in PD (GPi, SNr, and STN), which may be analogous to the functional inactivation of the STN or the GPi induced by DBS.

However, it is not possible to rule out the possibility that further brain structures containing GABA_A_ receptors, including specific neuron populations of some areas of the cerebral cortex, might be involved in the potential beneficial effects of zolpidem in improving Parkinsonian motor symptoms.

In a recent study on a mouse model of PD [37], it was shown that dopamine depletion induced by the neurotoxin MPTP may give rise (through D1 and D2 receptors) to marked changes in synaptic dynamics of pyramidal neurons in M1, which receive direct dopaminergic projections from the ventral tegmental area and the substantia nigra pars compacta. In such MPTP-treated mice, the synaptic remodeling in M1, induced through D1 and D2 receptors (with D1 receptor signaling linked to dendritic spine elimination and D2 receptor signaling linked to dendritic spine formation), gives rise to a net loss of stable dendritic spines in pyramidal cortical neurons of layer V and results in abnormal structural and functional plasticity in the motor cortex. Likewise, it was hypothesized that in patients with PD an abnormal synaptic plasticity in the motor cortex may contribute to the development of motor impairment (including impairment of motor learning) and that treatment strategies aimed at modifying such abnormal synaptic plasticity in the motor cortex may have beneficial effects in PD patients [37].

Recently, an experimental study in rats [38] using an* in vitro* brain slice model of neuronal oscillatory activity and a kinetic model of GABA_A_ receptor dynamics showed that zolpidem in low concentrations may reduce beta power in M1 cortical area, possibly through its action on inhibitory interneurons of the cortical area M1. Likewise, it has been also hypothesized [38] that in patients with PD low doses of zolpidem, through its possible effects on fast spiking inhibitory interneurons in M1 cortical area, may induce an increased tonic inhibition of such cortical interneurons in M1, which may give rise to an improvement of Parkinsonian motor symptoms.

## 4. Possible Mechanisms Underlying the Individual Variability of the Potential Effects of Zolpidem in Patients with PD

Current clinical evidence, although limited, suggests that, in those PD patients who show an improvement of Parkinsonian motor symptoms after the administration of zolpidem, the optimal dose of zolpidem may remarkably vary in different individuals. Similarly, the dosage of zolpidem which may induce sedation or drowsiness seems also to markedly vary in different PD patients, since some PD patients do not experience drowsiness even with relatively higher doses of zolpidem, while other PD patients do easily experience drowsiness after taking relatively low doses of zolpidem. On the whole, provided that there is apparently some individual variability of the effects of zolpidem in different patients affected by PD, the scanty available evidence suggests that in PD patients in more advanced disease stages (and presenting with more severe Parkinsonian motor symptoms) a single dose of zolpidem of about 10 mg (the standard dose for insomnia) is more likely to induce an improvement of Parkinsonian motor symptoms, in comparison with PD patients in early stages and presenting with mild Parkinsonian motor symptoms [18–22]. Likewise, PD patients in more advanced disease stages and presenting with more severe Parkinsonian motor symptoms seem on average to better tolerate doses of about 7.5 or 10 mg of zolpidem (namely, they show less frequently sedation or drowsiness after the assumption of such doses of zolpidem), as compared with PD patients in early stages and presenting with mild Parkinsonian motor symptoms [18–22].

It is not easy to understand why, at least in a subset of PD patients, zolpidem does not induce drowsiness at dosages which usually induce sleep in normal subjects and why there is a remarkable individual variability in the occurrence of an improvement of Parkinsonian motor symptoms after the administration of zolpidem. In the attempt to give a possible answer to such intriguing questions, it is helpful to take into account some prompts deriving from few experimental studies.

**Figure 2:**
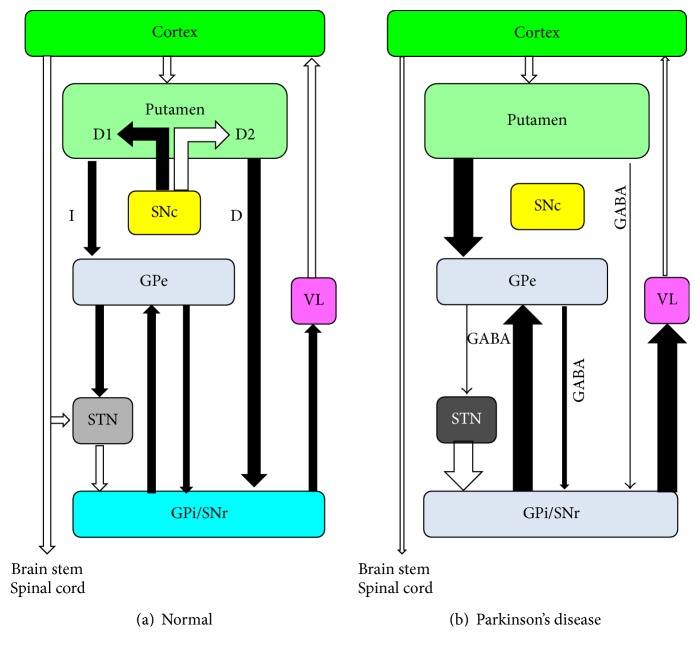
Schematic representation of the activity of the cortical-basal ganglia circuits in normal subjects (a) and in patients with Parkinson's disease/Parkinsonism (b), resulting in a possible compensatory upregulation of GABA_A_ receptors in various nuclei (STN, GPi, and SNr), which are overactive in PD. In PD, the underactivity of GABAergic neurons of the putamen and caudate projecting to GPi and SNr through the direct (D) pathway (b) may result in compensatory upregulation of GABA_A_ receptors [39] in deafferented brain structures, namely, the GPi and SNr (b). Similarly, we can hypothesize that in PD the decreased GABAergic inhibition exerted by the external globus pallidus (GPe) through the indirect (I) pathway on the glutamatergic neurons of the STN and the GABAergic neurons of the GPi and SNr (b) may result in compensatory upregulation of GABA_A_ receptors in such deafferented brain structures (STN, GPi, and SNr). Such compensatory upregulation of GABA_A_ receptors in deafferented brain structures (STN, GPi, and SNr) could be more marked in PD patients who, after the administration of zolpidem, show more evident beneficial effects on Parkinsonian motor symptoms and show no or minimal drowsiness. White arrows = excitatory connections; black arrows = inhibitory connections.

In animal models of Parkinsonism, after lesions of the substantia nigra pars compacta (SNc) or lesion of striatal nuclei, an increase of the density of GABA_A_ receptors containing the binding sites for BZ (previously named GABA_A_-BZ receptors) may occur in specific structures within the basal ganglia. In cynomolgus monkeys with experimental Parkinsonism induced by the injection of MPTP, an increased density of GABA_A_-BZ receptors was observed in the GPi [39]. After unilateral 6-hydroxydopamine lesions of the medial forebrain bundle in the rat, an increased number of GABA and BZ binding sites were detected in the SNr and in the entopenduncular nucleus, which is the rodent counterpart of the GPi [40]. Moreover, it has been suggested that in PD the underactivity of GABAergic neurons of the putamen and caudate projecting through the direct pathway to GPi and SNr may result in compensatory upregulation of GABA_A_ receptors [39] in deafferented brain structures, namely, the GPi and SNr (Figure 2(b)).

In normal subjects, homeostatic mechanisms in the STN [41] may regulate the balance between excitatory glutamatergic projections from the motor cortex to the STN and inhibitory projections from GPe to the STN (Figure 1(a)), while in PD patients such balance may be disrupted, resulting in an overactivity of the STN (Figure 1(b)). Recently, an experimental study [41] showed that in a model of PD in mice the overactivity of glutamatergic projections from the motor cortex to the STN may give rise not only to a pathologically increased activation of postsynaptic NMDA receptors in the STN (resulting in a pathological overactivity of the STN), but also to an increased expression of GABA_A_ receptors in the membrane of STN neurons.

On the basis of the experimental studies mentioned above [39–41], we can hypothesize that in PD the decreased GABAergic inhibition exerted by the external globus pallidus (GPe) through the indirect pathway on the glutamatergic neurons of the STN and the GABAergic neurons of the GPi and SNr may result in compensatory upregulation of GABA_A_ receptors in various deafferented brain structures, namely, the STN, GPi, and SNr (Figure 2(b)).

On the basis of such experimental studies [39–41], we might also speculate that, as compared to PD patients who after assuming zolpidem do not improve their motor symptoms or do experience drowsiness, in patients with PD who after the administration of zolpidem show an improvement of Parkinsonian motor symptoms and do not show remarkable drowsiness, a more marked upregulation of GABA_A_ receptors may occur in some critical structures overactive in PD (GPi, SNr, and STN). Accordingly, it might be hypothesized that in the subset of patients who resulted in being responders to zolpidem with a hypothetical more marked upregulation of GABA_A_ receptors in such overactive structures (GPi, SNr, and STN), zolpidem might bind to a relatively greater extent to these specific subcortical structures (GPi, SNr, and STN), at variance with other PD patients or normal subjects in whom zolpidem might preferentially bind to other brain structures (different from GPi, SNr, and STN) involved in sleep induction, thus inducing drowsiness. In the subset of PD patients responders to zolpidem, we might hypothesize that the relatively greater binding of zolpidem (Figure 1(c)) to such specific subcortical overactive structures in PD (GPi, SNr, and STN) may finally give rise to an increased inhibition of the two inhibitory output GABAergic structures of the basal ganglia (GPi and SNr), resulting in increased activity of specific thalamic nuclei and of specific cortical areas (such as the supplementary motor area). According to our hypothesis, such increased activity of specific cortical areas resulting from a more marked upregulation of GABA_A_ receptors in critical subcortical structures overactive in PD (GPi, SNr, and STN) might account for both the beneficial effects of zolpidem on Parkinsonian motor symptoms and the absence (or minimal degree) of drowsiness observed in a subset of PD patients (mostly with a good response to zolpidem), as compared to other PD patients who are nonresponders to zolpidem or show drowsiness even after low doses of this drug.

Accordingly, we might also speculate that, as compared to PD patients in early stages presenting with mild Parkinsonian motor symptoms, PD patients in more advanced disease stages and presenting with severe Parkinsonian motor symptoms might on average tolerate higher dosages of zolpidem, possibly because in advanced PD there might be a greater deafferentation of critical subcortical structures overactive in PD (GPi, SNr, and STN), in whom a more marked upregulation of GABA_A_ receptors may occur.

## 5. Potential Beneficial Effects of Zolpidem, Possibly through a Positive Allosteric Modulation of GABA_**A**_ Receptors Containing the ***α***1 Subunit, in Neurological Disorders other than PD

In the last two decades, following the initial serendipitous observation in PD [18], various reports have suggested that the administration of zolpidem may induce beneficial effects in a variety of neurological disorders other than PD, such as other Parkinsonian syndromes (as further briefly summarized below) and other neurological conditions associated with dysfunction of the basal ganglia, including dystonia [42–49] and tardive dyskinesia and acathisia [50]. In addition, it was reported that zolpidem may induce a symptomatic improvement in a variety of other conditions associated with motor dysfunction, such as restless leg syndrome [51], postanoxic spasticity [52], spinocerebellar ataxia [53], and central pontine myelinolysis [54]. Furthermore, it has been observed that the administration of zolpidem may induce beneficial effects in some patients with stroke [55] and in a subset of patients with disorders of consciousness [56–62].

Beneficial effects of zolpidem were reported in a variety of Parkinsonian syndromes other than PD, including Progressive Supranuclear Palsy [31, 63–66] and X-Linked Dystonia Parkinsonism [67, 68]. Although the beneficial effects on zolpidem on motor symptoms of Progressive Supranuclear Palsy (PSP) are not the main focus of this review, we will briefly mention some observations suggesting the possibility to improve motor symptoms (akinesia, rigidity, voluntary eye movements, dysarthria, and dysphagia) in patients with PSP, who are usually poorly responsive to levodopa and other anti-Parkinsonian drugs.

In the 90s, Daniele and coworkers observed a 58-year-old man affected by PSP with 5-year clinical history [31], who was given a 10 mg oral dose of an immediate-release formulation of zolpidem in the morning in the attempt to assess possible beneficial effects of zolpidem on motor symptoms of PSP, in analogy with the motor improvement previously observed in PD patients [18]. After the assumption of such single dose of zolpidem, this patient showed for some hours a remarkable improvement of several motor symptoms of PSP (akinesia, rigidity, voluntary eye movements, and dysarthria), in the absence of drowsiness. Following such observation, a pilot study (with a double-blind, placebo-controlled, crossover design) was carried out in 10 patients with a clinical diagnosis of probable PSP [31], who received in four separate trials in randomized order two single oral doses of zolpidem (5 and 10 mg), a single dose of levodopa (250 mg) plus carbidopa (25 mg), and placebo. After withholding of all anti-Parkinsonian medication 12 hours before motor assessment, PSP patients were examined by means of the UPDRS-III scale before (baseline) and one hour after the administration of the active drug or placebo. A positive response in each individual patient was defined as a decrease of more than 20% of the UPDRS-III score at baseline. In this pilot study, the administration of a single 5 mg dose of immediate-release zolpidem, unlike placebo, induced a slight but significant improvement of motor symptoms of PSP as assessed by UPDRS-III scale (namely, a 6.5% mean reduction of UPDRS-III scores from baseline). After the administration of zolpidem, two out of 10 PSP patients showed a decrease of more than 20% of the UPDRS-III score at baseline and an improvement of voluntary saccadic eye movements was observed in 4 out of 10 patients. In PSP patients responding to zolpidem, the motor improvement appeared 40–60 minutes after the administration of zolpidem, lasted about 2 hours, and mainly involved rigidity, akinesia/bradykinesia, and voluntary saccadic eye movements. In this pilot study, the only adverse effects observed after the administration of immediate-release zolpidem were drowsiness and increased postural instability, which were more marked after a single 10 mg dose of zolpidem. Subsequent observations with immediate-release and controlled-released formulations of zolpidem [63–66] confirmed that in patients with PSP the administration of zolpidem (both in single doses and chronically) may induce remarkable beneficial effects on most motor symptoms of PSP (akinesia, rigidity, voluntary eye movements, dysarthria, and dysphagia), which in some patients became more evident after the administration of controlled-released formulations of zolpidem, with a possible delay of some weeks between the start of treatment with controlled-released zolpidem and the appearance of an improvement of motor symptoms of PSP.

In 2001, Farver and Khan [34] described a 34-year-old man affected by schizoaffective disorder and antipsychotic-induced Parkinsonism with tremors of the hands for numerous years, unresponsive to various medications (benztropine, biperiden, amantadine, and propranolol). Zolpidem was administered to this patient (10 mg four times daily) and the tremor significantly decreased. Moreover, after one month of chronic treatment with zolpidem, the patient showed a dramatic improvement of Parkinsonian motor symptoms, with a score decrease from 29 at baseline to 9 on the motor examination part of the UPDRS (UPDRS-III). After a 4-month period of chronic treatment of the Parkinsonian motor symptoms of this patient with zolpidem (at a dosage of 10 mg q.i.d.), he showed a worsening of psychotic symptoms (delusions, hallucinations). Accordingly, clozapine was started and zolpidem was discontinued, due to excessive sedation induced by the administration of clozapine together with zolpidem. After the discontinuation of chronic treatment with zolpidem, tremor reemerged in this patient. In the attempt to improve again his Parkinsonian motor symptoms, chronic treatment with zolpidem was reinitiated at a lower dosage (5 mg q.i.d.), with stabilization of Parkinsonian motor symptoms over a 2-year period. This report suggests the potential benefits of the chronic administration of zolpidem in patients affected by Parkinsonism induced by neuroleptics.

In 2006, at the 10th International Congress of Parkinson's Disease and Movement Disorders organized by the Movement Disorders Society, Kawashima and coworkers [69] reported a 70-year-old woman with a diagnosis of Parkinsonism, presenting with resting tremor, bradykinesia, and rigidity. This patient was treated with levodopa/carbidopa, with unsatisfactory results, and underwent cardiac 123I-metaiodobenzylguanidine scintigraphy, which showed decreased uptake. Over the subsequent two years, Parkinsonian motor symptoms gradually progressed and at the age of 72 years she could not speak, eat, and walk. Fifteen minutes after the administration of a single 5 mg dose of zolpidem for insomnia, her rigidity and akinesia dramatically improved for about four hours and she could speak clearly, eat foods, and take several steps with bilateral assistance. After the administration of a single 5 mg dose of zolpidem, the total score obtained on the motor examination part of the UPDRS (UPDRS-III) decreased from 78 at baseline to 56. The authors suggested that, in a subgroup of patients with Parkinsonism with poor response to levodopa, the administration of zolpidem (even in low doses) may induce remarkable beneficial effects on Parkinsonian motor symptoms.

X-Linked Dystonia Parkinsonism (XDP), known otherwise as Lubag, is an X-Linked neurological syndrome afflicting male adults in the Philippines [68], clinically characterised by dystonia and Parkinsonism, poorly responsive to pharmacological treatment. XDP is associated with mutations in two genes, the /DYT3/ gene and the /TAF1/ (TATA binding protein-associated factor-1) gene. Dystonia usually starts focally in the lower limbs or oromandibular region and may then spread to become generalized, while Parkinsonism usually presents in later in the disease course, usually in combination with dystonia. It has been reported that the administration of a single 10 mg oral dose of zolpidem in three patients affected by XDP [67] induced after 15–45 minutes in all three patients a dramatic improvement of dystonia (100%, 32%, and 31% improvement on the Burke-Fahn-Marsden dystonia score, resp.) and a dramatic improvement of Parkinsonism (bradykinesia and rigidity) in 2 out of 3 patients (with 40%, 34%, and 0% improvement on the UPDRS-III scale, resp.). All three patients affected by XDP were treated chronically with zolpidem [67]. Patient 1, with a slow titration of the daily dose of zolpidem and with caffeine intake, adapted to sedation induced by zolpidem and was treated chronically with very high daily dosages of zolpidem (10 mg every 2 hours). On follow-up, one year after starting the chronic treatment with zolpidem, in patient 1, efficacy on motor symptoms of XDP was still maintained. Patient 2 did not tolerate daily doses of zolpidem higher than 20 mg but showed beneficial effects on motor symptoms of XDP after chronic treatment with a 20 mg daily dose of zolpidem (10 mg b.i.d.). Six months after starting the chronic treatment with zolpidem, patient 2 developed diarrhea and discontinued zolpidem, with subsequent disappearance of diarrhea. In patient 3, a chronic treatment with zolpidem had to be discontinued after 2 months, only due to financial straits of this patient. Since patient 3 was unemployed, a benefactor gave him 120 tablets of zolpidem, which he stretched out by taking only one 10 mg tablet b.i.d. In this patient, the administration of zolpidem 10 mg b.i.d. induced beneficial effects with no side effects, but he ran out of zolpidem tablets after 2 months of chronic treatment with zolpidem. In a review paper on XDP [68], it has been pointed out that zolpidem was the only drug showing remarkable beneficial effects on disabling motor symptoms of patients affected by XDP.

As to the mechanisms underlying the clinical improvement observed after the administration of zolpidem in various neurological disorders other than movement disorders (including Parkinson's disease and Parkinsonian syndromes), although such mechanisms are still unclear, it seems plausible to us that they might be related to the selective action of zolpidem on GABA_A_ receptors containing the *α*1 subunit, resulting in enhanced activity of specific GABAergic circuits across various brain structures. This general hypothesis is supported by a recent experimental study on ischaemic stroke in mice, suggesting that zolpidem may improve recovery after stroke in mice by enhancing phasic GABAergic inhibition during the repair phase of stroke [70], which can be associated with an increased number of *α*1 receptor subunit-containing GABAergic synapses in the peri-infarct cerebral cortex.

Currently available evidence [71–73] suggests that in patients with brain damage due to stroke or associated with various pathologies resulting in disorders of consciousness the likelihood of beneficial effects of zolpidem seems to be remarkably lower, unfortunately, as compared with the likelihood of a motor improvement in PD patients. In our view, in order to give a possible explanation for such relatively low likelihood of beneficial effects of zolpidem in patients affected by stroke or disorders of consciousness, we might hypothesize that in such neurological conditions the distribution of damage across different brain structures is much less predictable than in patients with PD, in whom the likelihood of beneficial effects of zolpidem seems accordingly to be relatively higher, consistently with the hypothesis of a selective action of zolpidem on GABA_A_ receptors of various subcortical structures (GPi, SNr, and STN), which are overactive in PD. In other words, in stroke or disorders of consciousness a poorly predictable distribution of damage across different brain structures results in high individual variability in the patterns of brain damage across different patients. Accordingly, only in a (possibly relatively small) subset of patients with stroke or disorders of consciousness, a positive allosteric modulation of GABA_A_ receptors induced by the administration of zolpidem may give rise to a positive allosteric modulation of GABAergic synapses in specific neuronal networks, which may result in a clinical improvement in such subset of patients. This hypothesis might account for those observations reporting that the proportion of brain-damaged patients with disorders of consciousness who improve after the administration of zolpidem might be relatively low in placebo-controlled and open-label trials carried out on a relatively higher number of subjects [71–73].

## 6. Concluding Remarks and Perspectives

Various studies and observations, although still preliminary and sometimes published only in abstract form, consistently suggest that zolpidem may induce beneficial (and often clinically remarkable) effects in the treatment of motor symptoms associated with a variety of movement disorders [35], including PD and other Parkinsonian syndromes.

As to PD, a potential role of zolpidem in the treatment of this disabling disorder is suggested by the following considerations: (a) a number of clinical observations provided some clear-cut, although preliminary, evidence that the oral administration of zolpidem may remarkably improve most motor symptoms of PD, even after a single dose of zolpidem, as may be observed only with the most powerful anti-Parkinsonian drugs (levodopa and apomorphine); (b) in patients with PD, unlike most anti-Parkinsonian drugs, zolpidem may also have antidyskinetic effects, which may be very helpful in patients with advanced PD, who experience dyskinesias and other symptoms associated with long-term levodopa treatment; (c) in patients with advanced PD, who become usually poorly responsive to current options of pharmacological treatment and in whom alternative pharmacological approaches are more urgently needed, the beneficial effects of zolpidem on Parkinsonian motor symptoms seem to be more remarkable and a lower incidence of side effects of zolpidem (such as drowsiness) may be observed with the standard 10 mg dose of zolpidem used for insomnia.

It remains to be ascertained whether in PD patients the possible beneficial effect of zolpidem on Parkinsonian motor symptoms may decrease after a certain period of treatment with zolpidem, possibly due to disease progression or due to the possible development of tolerance after the chronic administration of zolpidem. The scanty evidence available so far suggests that, at least in some individual PD patients, the chronic administration of zolpidem might have persistent beneficial effects for several months [21] or even for years, namely, more than 3 years [19] or up to 5 years [18]. Interestingly, in some PD patients the doses of zolpidem had not been substantially increased over more than 3 years of chronic administration [19] or had not been increased at all up to 5 years of chronic administration of zolpidem [18]. Long-term clinical trials are needed to clarify whether in PD patients the possible beneficial effects of zolpidem on Parkinsonian motor symptoms may decrease after a certain period of chronic treatment with zolpidem and to clarify whether specific clinical or pathophysiological features of PD patients might predict the occurrence over time of a decrease of beneficial effects of zolpidem on Parkinsonian motor symptoms.

The safety of a long-term treatment with zolpidem in patients with PD has been recently questioned by two recent retrospective observational studies, which analyzed a large Taiwan National Health Insurance database [74, 75]. These studies suggested that the overall incidence of PD was significantly greater among a group of subjects with a history of assumption of zolpidem for more than 3 months as sleep-inducer, as compared to a group of subjects without a history of assumption of zolpidem [74, 75]. In the first study [74], the group of subjects who received zolpidem had a higher cumulative rate of PD than the group of subjects who did not receive zolpidem during a 5-year follow-up period, while in the second study [75] after 5 years of observation the incidence of PD did not differ between the two groups. Such two studies, which according to their authors have various limitations, suggested the hypothesis that the assumption of zolpidem over more than 3 months may increase the risk for PD. Interestingly, some experimental studies had suggested that zolpidem may have neuroprotective effects, including* in vitro* and* in vivo* antioxidant activity [76–78] and protective effects against hypoxic stress [78]. It has been pointed out [79] that no plausible mechanism was proposed for the hypothetical relationship between the prolonged assumption of zolpidem and an increased risk for PD. An alternative and perhaps more parsimonious explanation for the findings of such two retrospective studies has been recently proposed by Andrade [79], who suggested that subjects with persistent sleep disturbances (and therefore assuming zolpidem for more than 3 months) are more likely to develop PD later, since it has been established that in patients who develop PD sleep disorders may antedate the onset of motor symptoms by several years [80]. According to this latter view, persistent sleep disturbances and the subsequent prolonged use of zolpidem may have some predictive value, as persistent sleep disturbances (requiring a prolonged use of zolpidem) in a subgroup of subjects may simply represent very early nonmotor symptoms of PD. Although according to this perspective the prolonged assumption of zolpidem per se would not play any significant role as a risk factor for PD, further epidemiological studies are needed in order to draw reliable conclusions about this potential safety issue related to a long-term treatment with zolpidem.

In conclusion, a number of questions and issues need to be faced at present. Controlled randomized clinical trials are certainly needed in order to confirm the efficacy of zolpidem in treating (possibly most) motor symptoms of PD patients, to investigate the safety and tolerability of zolpidem in PD patients, to find the most appropriate daily dose range of zolpidem that can be administered to PD patients (ideally, following an individualized and slow titration, in order to find the optimal daily dosage of zolpidem in each patient), to understand which are the best strategies to minimize the possible occurrence of daytime drowsiness as adverse effect of zolpidem, to check whether in patients treated with zolpidem plus levodopa (or other dopaminergic agents) it is possible to reduce the daily dosage of levodopa (or of other dopaminergic agents), and to identify potential features in individual PD patients (according to clinical symptoms or according to possible neurophysiological or neuroimaging markers) that might ideally predict a good chronic response to zolpidem in PD patients.
